# The impact of DNA extraction methods on species quantification and apparent community composition of *in vitro* oral biofilms

**DOI:** 10.1093/femsle/fnag066

**Published:** 2026-06-08

**Authors:** Jan-Ole Reese, Ingrid Maria Castro Lund, Håvard Jostein Haugen, Athanasios Saragliadis, Ståle Petter Lyngstadaas, Dirk Linke

**Affiliations:** Department of Biosciences, University of Oslo, 0316 Oslo, Norway; Department of Biosciences, University of Oslo, 0316 Oslo, Norway; Department of Biomaterials, Institute of Clinical Dentistry, University of Oslo, 0455 Oslo, Norway; Corticalis AS, Oslo Science Park, 0349 Oslo, Norway; Department of Biosciences, University of Oslo, 0316 Oslo, Norway; Department of Biomaterials, Institute of Clinical Dentistry, University of Oslo, 0455 Oslo, Norway; Corticalis AS, Oslo Science Park, 0349 Oslo, Norway; Department of Biosciences, University of Oslo, 0316 Oslo, Norway

**Keywords:** oral biofilm, peri-implantitis, *in vitro* multispecies model, implant surface, DNA extraction bias, genomic DNA extraction kits

## Abstract

*In vitro* multispecies biofilm models are widely used to study oral diseases and to evaluate treatment strategies for conditions such as peri-implantitis, often relying on accurate species quantification to assess treatment efficiency. However, the influence of DNA extraction methodology on downstream quantitative analysis has not yet been addressed for such model systems. Here, we evaluated three mechanistically distinct protocols, a custom phenol-chloroform approach, and two commercial kits employing different lysis strategies. These were applied to planktonic cultures of six peri-implantitis-associated species individually, as well as to defined multispecies biofilms grown on implant surfaces. Pure culture DNA yields differed substantially between methods, revealing pronounced species-dependent variation. Species-specific quantification of biofilm replicates by quantitative PCR resulted in community profiles that appeared dominated by either commensal early- or pathogenic late-colonizers, depending solely on the extraction approach employed. These findings demonstrate that DNA extraction is a critical yet often overlooked variable, capable of fundamentally altering the apparent community composition of *in vitro* biofilm models. Our work is intended to serve as a warning, emphasizing the need for method validation and standardization when applying DNA-based community profiling to biofilm models before drawing conclusions on relative species abundances.

## Teaser

In multispecies biofilm models, the choice of DNA extraction method is a key parameter for correct quantitative measurements of relative species abundance.

## Introduction

Peri-implant diseases represent one of the most prevalent complications in contemporary implant dentistry and constitute a major cause of implant failure (Derks and Tomasi 2015). Their pathogenesis is fundamentally driven by biofilm formation on the rough titanium implant surfaces, which triggers inflammatory responses that can progress to bone resorption and ultimately implant detachment (Berglundh et al. 2018, Fragkioudakis et al. 2021). Disease progression is closely associated with dysbiotic shifts within the biofilm microbiome, characterized by the enrichment of periodontal pathogens (Chen et al. 2025). Accurate, species-level identification and quantification of peri-implant biofilm communities are therefore essential for understanding disease pathogenesis and for evaluating preventive or therapeutic strategies.

To overcome the ethical and practical limitations of *in vivo* studies, sophisticated *in vitro* biofilm models have emerged as indispensable tools for investigating peri-implant infections. These systems are typically used to cultivate multispecies oral communities on implant-relevant materials to enable their systematic examination under controlled laboratory conditions (Ramachandra et al. 2023). Key aspects of the ecological succession observed *in vivo* are simulated by employing a few relevant oral species as representatives for established colonization patterns, with common consortia comprising between four and ten proxy species (Guggenheim et al. 2009, Sánchez et al. 2011, Kommerein et al. 2017, Ramachandra et al. 2023). Such models serve as platforms to investigate colonization patterns and spatial biofilm organization (Thurnheer et al. 2004), while also facilitating preclinical evaluation of surface modifications or debridement and decontamination strategies (Kommerein et al. 2017, Bravo et al. 2023, Hussain et al. 2024).

Precise interpretation of these *in vitro* model systems, analogous to *in vivo* microbiome studies, increasingly relies on molecular quantification methods, particularly quantitative PCR (qPCR) and next-generation sequencing (NGS). Correct abundance estimates are crucial for characterizing community composition and for detecting ecological shifts, such as altered ratios between health-associated and pathogenic taxa following surface modifications. Similarly, assessment of treatment efficacy (e.g. by monitoring pathogen reduction) depends on accurate quantitative readouts. Despite this growing reliance on molecular methods, remarkably little attention has been paid to whether DNA extraction, the critical first step in molecular workflows, provides an accurate and representative sample for the quantitative analysis of defined *in vitro* biofilm consortia. Currently, there is no consensus on a standard DNA extraction method and, to our knowledge, no systematic evaluation has examined the reliability of existing approaches in capturing the true community composition of these defined biofilms.

It is increasingly recognized in microbiome research in general that systematic bias can produce discrepancies between observed and true microbial community composition (Brooks 2016). Such discrepancies have been well documented for common analytical methods, including qPCR, Whole MetaGenome Shotgun Sequencing, and 16S rRNA sequencing (Wesolowska-Andersen et al. 2014, Brooks et al. 2015, Lima et al. 2022). Among all steps contributing to this bias, genomic DNA extraction has been determined as a key driver for experimental variability (Greathouse et al. 2019, Rauer et al. 2025), prompting large-scale benchmarking efforts by the MicroBiome Quality Control (Sinha et al. 2017) and the International Human Microbiome Standards project (Costea et al. 2017).

Consequently, several studies have addressed the influence of different DNA extraction methods on various microbiomes and applications. These investigations typically compare commonly used commercial extraction kits, which generally rely on DNA binding to a solid matrix, but also often include more traditional approaches employing phenol-chloroform for organic DNA separation. Various studies have evaluated the DNA extraction bias in natural samples from diverse ecosystems, including environmental ones (Walden et al. 2017, Liu et al. 2019, Mateus-Barros et al. 2019, Stojan et al. 2023), as well as the human microbiome (Kennedy et al. 2014, Wesolowska-Andersen et al. 2014, Mattei et al. 2019, Rehner et al. 2022). Most of these studies report substantial variation in DNA yield and purity, as well as species richness and abundance, across methods. However, evaluating which method yields the most accurate community composition estimates downstream remains challenging, as the true composition is typically unknown. To address this limitation, numerous studies employed mock communities containing known proportions of bacterial species as representatives for specific microbiomes (Morgan et al. 2010, Yuan et al. 2012, Brooks 2016, Costea et al. 2017). These studies have demonstrated that DNA extraction distorts taxonomic community composition, with variation both among extraction methods and relative to expected abundances.

Incomplete and differential lysis of bacterial cells has been identified as a major contributor to this extraction bias (Li et al. 2020, Karstens et al. 2021). This predominantly affects “hard-to-lyse” species, particularly Gram-positive species whose rigid cell walls provide mechanical resistance (Costea et al. 2017), often shifting the apparent community composition in favour of more readily lysed Gram-negative species (Karstens et al. 2021). Additionally, the release of genomic DNA from biofilm-associated bacteria poses particular challenges due to their organization within an extracellular polymeric substance (EPS) matrix (Flemming et al. 2016). Beyond providing mechanical stability and protection against lysis, EPS components, such as polysaccharides, lipids, and proteins, are known to bind DNA during extraction, thereby impeding DNA isolation and introducing additional contaminants (Corcoll et al. 2017, Govil et al. 2019).

Controlled *in vitro* biofilm models are increasingly employed as reference systems for implant research, yet they may be particularly vulnerable to DNA extraction bias: These models typically contain only few species, which possess known differential lysis susceptibilities and are embedded in protective EPS matrices that add to lysis resistance. In such defined consortia comprising limited species diversity, even modest extraction biases affecting individual taxa can have a pronounced effect on apparent relative community profiles, compared with taxonomically rich *in vivo* biofilms. Few comparison studies have specifically addressed DNA extraction efficiency from microbial biofilms (Hwang et al. 2012, Corcoll et al. 2017, Govil et al. 2019), and studies focusing on oral biofilms are limited to *in vivo* samples such as clinical supragingival plaque (Abusleme et al. 2014, Vesty et al. 2017, Wäge-Recchioni et al. 2025). DNA extraction is often treated as a routine step when examining *in vitro* models, and to our knowledge, no studies have evaluated the influence of DNA extraction methodology on these systems.

In the present study, we address this methodological gap by systematically evaluating how three different DNA extraction procedures (comprising a custom phenol-chloroform protocol and two commercial kits widely employed for oral biofilm samples) affect yield, purity, and species-specific community composition in a defined *in vitro* oral biofilm model. Six peri-implantitis-associated bacterial species, representing early (*Streptococcus oralis, Actinomyces naeslundii)*, intermediate (*Veillonella parvula, Fusobacterium nucleatum*), and late/pathogenic (*Porphyromonas gingivalis, Aggregatibacter actinomycetemcomitans*) colonization stages, were examined as both planktonic pure cultures and multispecies biofilms grown on implant-like titanium surfaces. Direct species-resolved quantification was performed by *rpoB*-targeted qPCR. By comparing three mechanistically distinct DNA extraction approaches, we aimed to quantify the extent to which extraction methodology introduces systematic bias into both total species abundances and community profiling of model oral biofilms. Although often overlooked, accurate determination of absolute microbial abundances is essential for applications ranging from biofilm biomass estimation to precise diagnostics and treatment monitoring, and will become increasingly relevant for future systems biology models requiring exact quantitative inputs. Our findings have direct implications for the interpretation of microbiome data derived from *in vitro* biofilm models and, more broadly, for the comparability of results across studies employing such models with varying DNA extraction strategies.

## Material and methods

### Bacterial strains and culture conditions

Bacterial species used in this study are common representatives of different stages involved in the development of oral biofilms, including both Gram-positive and Gram-negative organisms. The composition of the consortium derived from previous publications (Blanc et al. 2014, Hussain et al. 2024) and all strains were obtained from the respective labs. Strains were as follows: *S. oralis* NCTC 11427, *A. naeslundii* ATCC 19039, *V. parvula* NCTC 11810, *F. nucleatum* DSM 20482, *P. gingivalis* ATCC 33277, and *A. actinomycetemcomitans* DSM8324. As described in Reese et al. (2026), all species were first cultivated anaerobically on blood agar plates (Blood Agar Base No. 2, VWR Chemicals, Leuven, Belgium), supplemented with 5% (v/v) defibrinated sheep blood (Thermo Scientific Oxoid, Basingstoke, UK), 5.0 mg L^−1^ hemin (Fisher Scientific, Loughborough, UK), and 0.5 mg L^−1^ menadione/vit. K_3_ (Sigma-Aldrich, St. Louis, MO, USA).

### Preparation of pure planktonic cultures

Planktonic cultures were obtained as detailed in Reese et al. (2026). In summary, individual species were cultivated in modified brain-heart infusion medium (BHI), containing 37 g L^−1^ BHI broth (VWR Chem.), 5.0 mg L^−1^ hemin (Fisher Sci.), 2.5 g L^−1^ mucin (porcine, type II), 1 g L^−1^ yeast extract, 2 g L^−1^ sodium bicarbonate, 0.5 mg L^−1^ menadione/vit. K_3_, 0.5 g L^−1^ cysteine, and 0.1 g L^−1^ glutamic acid (all Sigma). Strict anaerobic conditions were achieved by employing a Widdel flask and following the standard Hungate technique, as described by Widdel (1980). The pH of the bicarbonate-buffered medium was equilibrated by flushing with a gas mixture of N_2_/CO_2_ (95:5, v/v). Liquid cultures were incubated anaerobically at 37°C for 24–48 h, and growth was monitored spectrophotometrically (λ = 600 nm; BioPhotometer, Eppendorf AG, Hamburg, Germany). Cultures were harvested by centrifugation (5000 × *g*, 10 min) at an optical density (OD_600_) of 0.8. Each culture was divided into 500 µL aliquots, and the cell pellets were stored at −20°C for 3–6 days before DNA extraction.

### Preparation of titanium-based surfaces

For the static biofilm model, grade II titanium discs (provided by the mechanical workshop at the Faculty of Medicine, University of Oslo, Norway) with a surface area of 30 mm^2^ featuring a clinically relevant rough topography, were used to simulate peri-implantitis. Surface modifications followed the grit-blasting and acid-etching procedure described by De Lauretis et al. (2025b) to produce rough surface characteristics, comparable to commercial OsseoSpeed® dental implants (Dentsply Sirona, Charlotte, USA). The resulting topography exhibited moderate roughness, with a *Sa* of ~2.1 µm, effective fluid retention in the core region (*Sci* ≈ 1.5), negative skewness (*Ssk* ≈ −0.3), and high kurtosis (*Sku* ≈ 4) (De Lauretis et al. 2025a, Wang et al. 2025). Prepared discs were stored in ethanol at ambient temperature.

### Preparation of static multispecies oral biofilms

Static multispecies biofilms were established as described in Reese et al. (2026). In summary, treated titanium discs were placed within the wells of a nontreated, polystyrene 24-well tissue culture plate (VWR International, Leuven, Belgium) and the modified BHI medium described earlier was employed to cultivate oral biofilms on these implant-like surfaces. All six individual strains were grown under anaerobic conditions as described above until mid-exponential phase and then pooled to create a mixed-species inoculum. Species ratios of the inoculum were adapted from Sánchez et al. (2011) and proportionally adjusted to achieve an initial OD_600_ of 0.1 in the mixed culture. The wells, equipped with titanium discs, were filled with 1.5 mL mixed bacterial culture and incubated under an anoxic atmosphere of N_2_/CO_2_ (95:5, v/v) for 84 h.

For biofilm harvesting, titanium discs were gently rinsed in sterile PBS to remove unattached cells. Sample discs were then transferred into 500 µL sterile PBS and biofilm detachment was facilitated by vigorous vortexing (1 min), followed by ultrasonication (20 min, 37°C). After removing the discs, biofilm samples were stored at −20°C for 3–6 days before DNA extraction.

### DNA extraction

For genomic DNA extraction, three different methods were employed. Each extraction method was applied to three independent aliquots from each pure culture to obtain technical replicates (*n* = 3 per species). For biofilm samples, which could not be brought into a homogenous suspension, biological replicates were generated (*n* = 3). Each method was used to extract DNA from three separate multispecies biofilms from the same experiment. Equal volumes of 100 µL were used to elute the isolated DNA at the end of each extraction protocol to yield comparable DNA concentrations. Concentration and purity were determined using a NanoDrop ND-1000 spectrophotometer (Thermo Fisher Scientific, DE, USA).

### Method 1 (phenol-chloroform extraction)

The phenol-chloroform approach was based on the protocol described in Reese et al. (2026). Cell lysis was achieved through three freeze/thaw cycles, followed by enzymatic lysis. Samples were incubated with lysozyme (20 mg/mL final conc.) in lysis buffer containing 20 mM Tris·HCl (pH 8.0), 2 mM EDTA, and 1.2% Triton X-100, at 37°C for 30 min. Protein digestion was performed using Proteinase K (1 mg/mL final conc.) in complete lysis buffer (100 mM Tris-HCl, pH 8.0, 20 mM EDTA, 200 mM NaCl, 2% SDS) at 56°C for 1 h, followed by RNAse A treatment (10 µg/mL final conc.) at 37°C for 1 h.

DNA was isolated from the lysate by mixing with an equal volume of phenol:chloroform:isoamyl alcohol (25:24:1; saturated with 10 mM Tris, 1 mM EDTA, pH 8.0). The suspension was transferred to a pre-sterilised tube containing 200 µL polydimethylsiloxane/silicone-dioxide gel (Dow Corning, MI, USA) (Mukhopadhyay and Roth 1993). Phases were separated by centrifugation (16 000 × *g*, 5 min). The aqueous phase was reextracted accordingly with one volume of chloroform:isoamyl alcohol (24:1) to remove potential phenol residues. DNA was then precipitated by adding 1/10 volume of 3 M sodium acetate, 1 µL of glycogen (20 µg/µL), and 2.5 volumes of ice-cold absolute ethanol. After overnight incubation at −20°C, the precipitated DNA was collected by centrifugation (16 000 × *g*, 30 min, 4°C) and subsequently washed twice with 70% ethanol, air-dried, and resuspended in 100 µL of 10 mM Tris-HCl (pH 8.0).

### Method 2 (commercial kit—blood and tissue)

DNA extraction was carried out using the Qiagen DNeasy Blood and Tissue Kit (Qiagen, Hilden, Germany). The standard extraction protocol was adjusted, following manufacturer’s instructions to include a pretreatment for Gram-positive and Gram-negative bacteria. This included cell lysis by a combination of chemical and enzymatic lysis: Samples were incubated (30 min, 37°C) in an enzymatic lysis buffer containing 20 mg/mL lysozyme, 20 mM Tris·HCl (pH 8.0), 2 mM EDTA, and 1.2% Triton X-100, followed by incubation (60 min, 56°C) with Proteinase K in a supplied lysis buffer. Subsequent purification employed the provided silica spin-columns (*DNeasy Mini spin*) and eventual elution with 100 µL provided elution buffer.

### Method 3 (commercial kit—PowerBiofilm)

DNA extraction was carried out using the DNeasy PowerBiofilm Kit (Qiagen, Hilden, Germany), following manufacturer’s instructions. The kit achieved cell lysis by chemical lysis buffer (detergent) and heat treatment (65°C, 5 min.) followed by mechanical bead beating, using the supplied *PowerBiofilm Bead Tubes*. DNA binding and washing were facilitated through provided silica spin-columns (*DNeasy Mini spin*), followed by elution with 100 µL provided elution buffer.

### Species-specific quantification by qPCR

The abundance of each individual species within the extracts from multispecies biofilms was assessed by qPCR, targeting the single-copy RNA polymerase beta-subunit gene (*rpoB*) (Ogier et al. 2019). A species-specific primer set to amplify regions between 114 and 198 bp (Supplementary Table 1) was used as described previously (Reese et al. 2026). Each qPCR reaction was performed in 10 µL final volume, containing 5 µL SYBR qPCR Master Mix (VWR, Leuven, Belgium), 0.25 µL forward/reverse primer (10 µM), 2 µL template DNA, 2% dimethyl sulfoxide (DMSO), and rest nuclease-free water. Samples were subjected to initial denaturation at 95°C for 15 min, followed by 35 cycles of denaturation at 95°C for 15 s and annealing/extension at 66°C for 30 s with fluorescence acquisition. Primer specificity was verified by single peaks during final melt curve analysis (66°C–95°C with 0.2°C increments). All DNA samples were quantified in technical duplicates and no-template controls included in each run. Analysis was performed using a LightCycler® 96 System (Roche, Switzerland).

Standards for all target species were prepared from the pure culture extractions, obtained with the three different extraction methods. Method-specific standard curves were generated for each extraction method using five-fold dilutions of genomic DNA (5 × 10^−8^ to 1.6 × 10^−11^ g). Biofilm samples were run alongside the standard curves derived from their corresponding extraction method for quantification. Quantification cycle (C_q_) values and their correlation to DNA amount from standard curves were determined using the internal LightCycler^®^ 96 Application Software (V1.1, Roche Diagnostics).

### Statistical analysis

Data plotting and statistical analysis were performed using the software Origin2022b (OriginLab Corp., MA, USA) and StataSE 17 (StataCorp, TX, USA). Normal distribution of replicates within the three different methods was assessed by Shapiro–Wilk test. Homogeneity of variance across methods was evaluated with Levene’s test. One-way ANOVA was conducted separately for each species to assess significance of differences across all methods. Post-hoc analysis by the Tukey HSD test was used to compare the three tested extraction methods for each species. The statistical significance was set at *P* < .05 for all analyses.

## Results and discussion

For this study, we compared three DNA extraction methods to evaluate their suitability for investigating oral *in vitro* biofilm communities: A customized phenol-chloroform-based protocol and two commercial silica-matrix-based extraction kits with distinct lysis strategies that are widely utilized in oral microbiome research. Traditional phenol-chloroform isolation (Method 1) typically achieves higher total DNA yields compared to silica-matrix-based methods (Rosenbaum et al. 2019, Molbert et al. 2023). Here, it was combined with a rigorous lysis protocol optimized for maximal and uniform lysis across diverse bacterial morphotypes, as well as silicone gel-mediated phase separation. Due to its slightly higher density than water, the silicone gel accumulates between the organic and aqueous phases, forming a barrier layer that facilitates the clean recovery of the entire aqueous phase (Mukhopadhyay and Roth 1993). The commercial DNeasy Blood and Tissue kit (Method 2) relies on a chemical lysis approach, is commonly employed in periodontal biofilm studies and includes manufacturer-validated protocols for both Gram-negative and Gram-positive bacteria (Wäge-Recchioni et al. 2025). The commercial DNeasy PowerBiofilm kit (Method 3) incorporates mechanical bead beating lysis, which has been reported to improve DNA extraction from resilient Gram-positive species, including *A. naeslundii* (Li et al. 2020). Designed specifically for biofilm samples, this kit has previously been employed for metagenomic analysis of oral biofilm consortia similar to the one examined in the present study (Muras et al. 2020). The performance of each method was initially assessed with pure planktonic cultures of six relevant oral species to determine species-specific DNA recovery. The extraction methods were subsequently applied to defined multispecies biofilms cultivated on titanium surfaces for 84 h, where extracellular matrix components and structural complexity pose additional challenges for DNA extraction.

### Influence of extraction method to DNA yield from pure cultures

**Figure 1:**
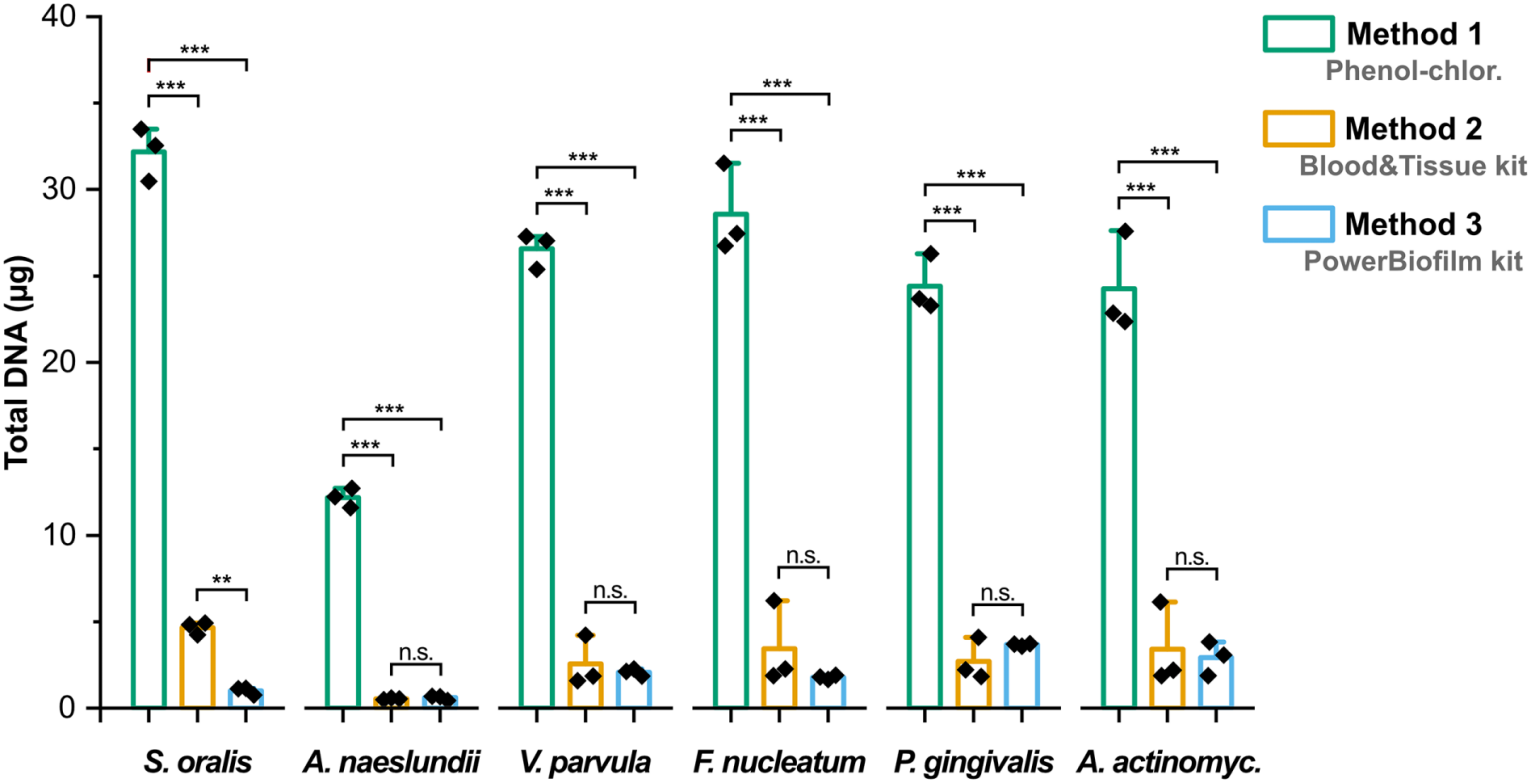
Comparison of total DNA yields from single-species suspension cultures of oral bacteria. Total DNA (ng) recovered from single-species planktonic cultures, using three extraction methods: Method 1 (Phenol-Chloroform; left/green bars), Method 2 (Commercial Kit—Blood & Tissue; middle/orange bars), and Method 3 (Commercial Kit—PowerBiofilm; right/blue bars). Pure cultures of oral bacteria were harvested at OD_600_ = 0.8 and aliquoted into technical replicates for each extraction method. Data represent mean values of technical replicates (◆; *n* = 3). Statistical differences between methods were assessed for each species by one-way ANOVA followed by Tukey’s HSD test (statistical analysis shown in Supplementary Tables 3–5). Significance: ***P < .001, **P < .01, *P < .05, n.s. = not significant.

The extraction of pure planktonic cultures revealed clear differences in the performance of the three methods (Fig. 1). Method 1 consistently achieved the highest DNA yields across all six species, significantly outperforming both other methods (*P* < .001). Total DNA recovery with this method ranged from ~24 to 32 µg across all species, except *A. naeslundii*, which yielded an average of ∼12 µg. Using the identical starting material, both methods employing commercial kits yielded substantially lower amounts (∼0.5–5 µg), with comparable performance across all species except *S. oralis*, where Method 2 yielded significantly more than Method 3 (*P* < .01).

Species-specific patterns emerged within each extraction method. Although differences in cell density at equivalent optical density do not permit direct comparison of DNA yields between species, the disproportionate intra-species variations between methods clearly indicate that extraction performance is not uniform across species. For *A. naeslundii*, total yields were ~55% lower than the mean of the other five species with Method 1, whereas they were 84% and 74% lower with Methods 2 and 3, respectively (Supplementary Table 2). The other Gram-positive species tested, *S. oralis*, achieved the overall highest total DNA in Methods 1 and 2. Conversely, it yielded among the lowest amounts with Method 3, where *P. gingivalis* presented the highest recovery instead.

A significantly higher DNA recovery with phenol-chloroform extraction compared to methods based on commercial kits has previously been demonstrated with oral samples by Rosenbaum et al. (2019), who suspected DNA loss during silica column purification. The overall superior yield achieved with Method 1 in this study can likely be attributed to the comprehensive cell lysis strategy, while minimizing DNA loss during purification. This was accomplished through a combination of mechanical disruption by freeze/thaw cycles with chemical and enzymatic lysis, followed by highly efficient nucleic acid extraction using phenol. The prevention of DNA loss during this step, which is crucial for quantitative approaches, was addressed by incorporating a phase-separating gel that permits recovery of the entire (DNA-containing) aqueous phase. The protocol has been optimized particularly to target the resilient cell structures of *A. naeslundii*, which possesses a unique cell wall composition (Schleifer and Kandler 1972), broad resistance to lytic enzymes (Delisle et al. 2006), and is considered a hard-to-lyse species (Aas et al. 2005). Biesbroek et al. (2012) described a higher prevalence of Gram-positive bacteria in saliva samples when mechanical disruption by bead beating was employed for DNA extraction. In contrast, we observed that both Gram-positive species (*S. oralis* and *A. naeslundii*) exhibited the lowest yield when extracted using Method 3, which involved lysis by bead beating. These results align with those of Abusleme et al. (2014) and Rosenbaum et al. (2019), who demonstrated lower DNA yields from both defined oral species and natural oral samples, respectively, when bead beating was applied as opposed to chemical and enzymatic lysis. The particular challenge posed by *A. naeslundii* was further highlighted by Li et al. (2020), who reported that an enhanced bead beating protocol was required for an adequate DNA extraction in this species. The additional mechanical disruption, in turn, compromised DNA recovery from Gram-negative oral bacteria in their study.

Extraction efficiencies could not be quantitatively assessed since the biofilm-forming bacteria examined in this study formed persistent multicellular aggregates, even in planktonic cultures. Although various measures, such as treatment with ultrasonication and detergents, were employed to disperse these aggregates, accurate cell enumeration by either CFU plating or direct microscopic counting did not yield reproducible results. The completeness of recovery with Method 1, which achieved the highest absolute DNA quantities for all species, can therefore not be assessed, as the theoretical total DNA content per sample could not be calculated. While this limitation must be acknowledged, it does not diminish the key finding of distinct species-specific extraction patterns observed with pure cultures for each method. The choice of method could thus systematically introduce bias not only in total amounts but also in the apparent relative abundance of species when examining polymicrobial samples.

### DNA purity

To ensure sufficient purity for subsequent qPCR measurements, the DNA quality of all samples was evaluated using spectrophotometric absorbance ratios (Table 1). Historically, the use of these ratios stems from observations that nucleic acids and (aromatic) amino acids exhibit distinct UV absorption maxima near 260 nm and 280 nm, respectively (Warburg and Christian 1942, Layne 1957), allowing the ratio of both values to be used for assessing protein contamination in DNA samples and vice versa (Manchester 1996). Standard quality thresholds consider A_260_/A_280_ ratios of 1.8–2.0 as pure DNA, largely free from protein contamination, while A_260_/A_230_ ratios ≥ 1.8 reflect the absence of common contaminants such as salts, carbohydrates, or phenol (Olson and Morrow 2012). The A_260_/A_280_ ratios remained within the accepted range for all pure culture extractions across all methods and for mixed-species biofilm extractions using Methods 1 and 2 (1.87–2.04). Biofilm samples processed with Method 3 yielded less pure DNA, with an A_260_/A_280_ ratio of 1.66 ± 0.06 slightly below the acceptable threshold, suggesting minor protein contamination. This biofilm-specific protein carryover likely stems from abundant EPS-associated extracellular proteins (Flemming et al. 2016), potentially exceeding the kit’s washing/removal capacities. The enzymatic protein digestion employed in both other methods likely prevents this issue.

**Table 1:** Average DNA yield and quality of all six single-species extractions and the biofilm extractions for all tested methods.

	Combined pure culture extractions			Biofilm extractions		
	Method 1	Method 2	Method 3	Method 1	Method 2	Method 3
**Total DNA yield (µg)**	24.7 ± 3.93	2.89 ± 0.79	2.01 ± 0.66	11.99 ± 0.12	2.54 ± 0.1	3.03 ± 0.44
DNA Purity						
**Abs 260 nm/280 nm**	1.96 ± 0.02	1.90 ± 0.06	1.93 ± 0.05	2.04 ± 0.02	1.87 ± 0.03	1.66 ± 0.06
**Abs 260 nm/230 nm**	1.50 ± 0.07	1.29 ± 0.15	0.74 ± 0.29	1.86 ± 0.09	1.17 ± 0.08	0.55 ± 0.35

Total yield in µg and DNA purity (absorbance ratios of 260/280 nm and 260/230 nm) were determined by NanoDrop measurements. All values are given as the arithmetic mean ± standard error of the mean. For pure cultures: Mean across all technical replicates from six separate single-species extractions (*n* = 18 total: 3 replicates of 6 species). For biofilms: Mean from multispecies biofilm extractions (*n* = 3 biological replicates).

The analysis of A_260_/A_230_ ratios revealed a greater variation across methods: Method 1 consistently achieved the highest purity across all samples, whereby only the biofilm extractions (1.86 ± 0.09) exceeded the recommended threshold. Pure culture extractions yielded slightly lower values (1.50 ± 0.07), possibly indicating residual phenol carryover despite gel-mediated phase separation, which could be mitigated by an additional chloroform washing step. Method 2 showed moderate purity ratios for pure culture and biofilm extractions (1.29 ± 0.15 and 1.17 ± 0.08, respectively), while Method 3 exhibited the lowest purity ratios (0.74 ± 0.29 and 0.55 ± 0.35, respectively). The reduced A_260_/A_230_ ratios observed with both commercial kits potentially result from residual guanidine salts, chaotropic agents employed by both methods, that are known to cause this issue (Koetsier and Cantor 2019). Residual polysaccharides, ethanol, or EDTA could additionally reduce these ratios across all methods. Despite lower purity scores in a subset of samples, all extracted DNA samples performed adequately in downstream qPCR assays. This is consistent with observations that the A_260_/A_280_ ratio, which remained within acceptable limits across all our methods, is a more crucial indicator determining the suitability of PCR applications (Olson and Morrow 2012).

### The choice of extraction method determines species-specific DNA yield from polymicrobial biofilms

The six species used for pure culture extractions were combined to cultivate polymicrobial biofilms under static conditions. The resulting bacterial consortium comprised typical representatives of oral biofilms and reflected the characteristic colonization pattern associated with implant-related supragingival infections. Similar biofilm consortia have been employed in established *in vitro* models, including those developed by Sánchez et al. (2011) and Blanc et al. (2014). Using Method 1 to isolate DNA from these biofilms resulted in a total yield of 11.99 ± 0.12 µg (Table 1), about four-fold higher than the yields obtained with Methods 2 and 3 (2.54 ± 0.1 µg and 3.03 ± 0.44 µg, respectively). The abundance of each species within these isolates was assessed by qPCR using primers targeting the *rpoB* gene. The single-copy nature of this housekeeping gene allows for accurate species discrimination while avoiding the abundance bias of multicopy genes (e.g. 16S rRNA) (Ogier et al. 2019).

**Figure 2:**
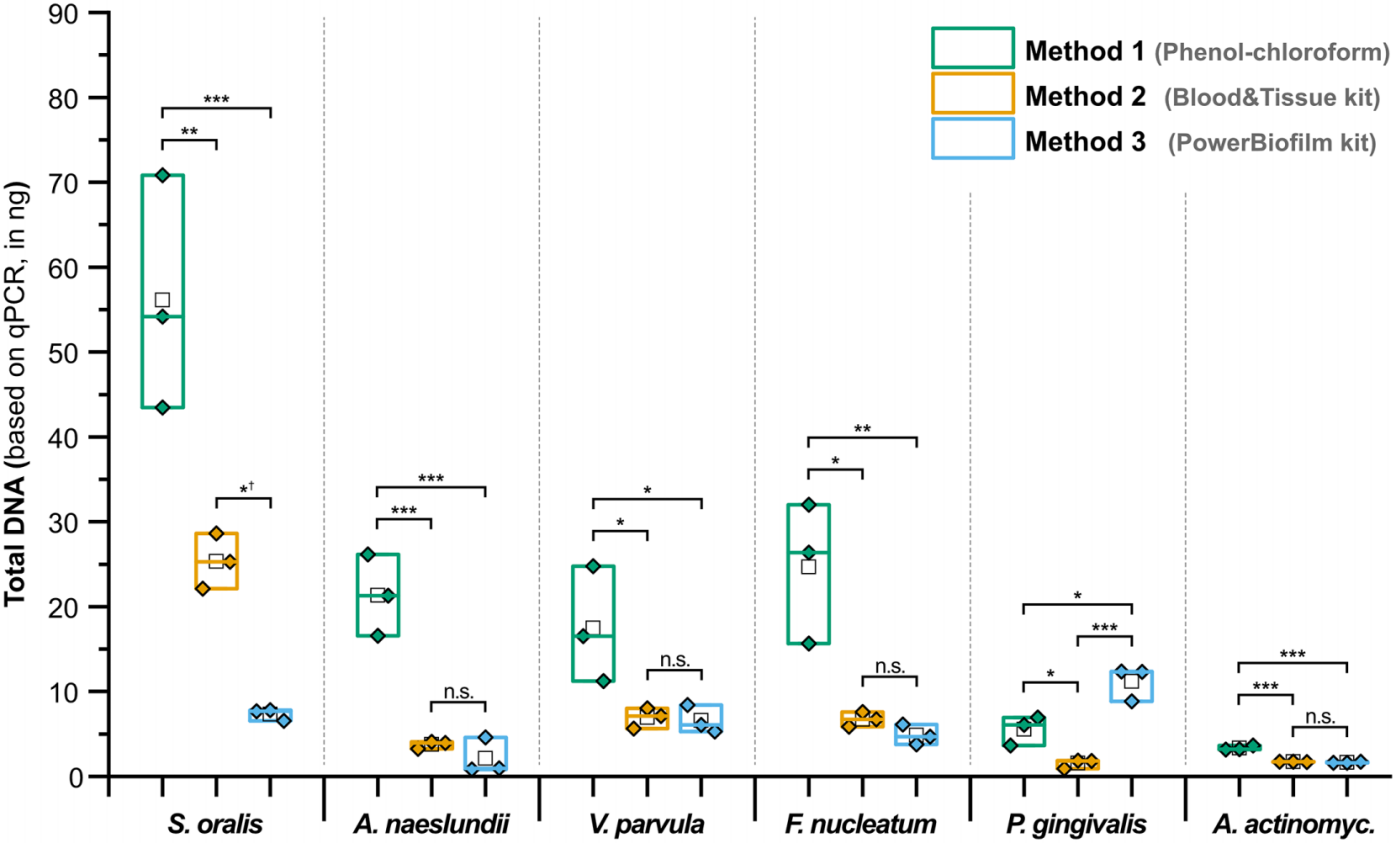
The influence of extraction methods on qPCR-based total DNA amount recovered from individual species in multispecies biofilms. Absolute quantification of DNA content (in ng) per species from six-species biofilms, comparing three extraction methods: Method 1 (Phenol-Chloroform; green), Method 2 (Commercial Kit—Blood and Tissue; orange), and Method 3 (Commercial Kit—PowerBiofilm; blue). Biofilms were cultivated anaerobically under static conditions on Ti-discs for 84 h. Quantification of individual species was performed by qPCR, using species-specific primers targeting the single-copy *rpoB* gene. Conversion of C_q_ to DNA mass (in ng) was based on individual standard curves specific to species and method. Box plots show median (line), quartiles, mean (□), and individual replicates (◆; *n* = 3 biological replicates). Statistical differences between methods were assessed for each species by one-way ANOVA followed by Tukey’s HSD test (statistical analysis shown in Supplementary Tables 6–8). Significance: ***P < .001, **P < .01, *P < .05, n.s. = not significant. ^†^ Note: The *S. oralis* M2 vs. M3 comparison approached significance by Tukey’s HSD (*P* = .08) but was confirmed significant by supplementary two-sample t-test (*P* = .001).

The analysis of species abundance, based on DNA quantification using qPCR (Fig. 2) revealed a pattern that largely aligns with the one observed for pure culture extractions (Fig. 1). Method 1 consistently showed the highest absolute DNA yields, but the relative differences to the other methods varied strongly from species to species. The most pronounced difference was observed for *A. naeslundii*, with Method 1 yielding approximately 6- and 10-fold higher total abundances compared to Methods 2 and 3, respectively. *Streptococcus oralis* was by far the most abundant species according to Methods 1 and 2, but with significant differences in absolute yield, while Method 3 results suggest that *P. gingivalis* is the most abundant species in the biofilm model (in line with extraction yields obtained from pure cultures). This also represents the sole instance in which a commercial kit outperformed the phenol-chloroform-based protocol.

In comparison to the planktonically grown pure cultures, the arrangement of cells within polymicrobial biofilms introduces additional complexity: Bacterial cells are embedded within an EPS matrix that provides mechanical stability, restricts the dispersion of cells and offers protection against lytic agents (Pinto et al. 2020, Jakubovics et al. 2021, Li et al. 2023). Spatial stratification within the biofilms and strong cell-cell adhesion forces create compact microcolonies that exhibit greater mechanical resistance to disaggregation (Kaplan 2010, Zijnge et al. 2010, Bowen et al. 2018), potentially impairing the effectiveness of lysis protocols. In addition, Confocal and Atomic Force Microscopy studies of oral biofilms demonstrated that the cell-cell adhesion, as well as the matrix volume and stiffness, increase with continuing biofilm maturation (Bowen et al. 2018, Li et al. 2023). These observations suggest that the DNA extraction-related challenges documented here may be even more pronounced in biofilms subjected to extended maturation periods, as commonly encountered in clinical samples or long-term *in vitro* models.

Despite the increased sample complexity, the species-specific extraction patterns observed in pure cultures were recapitulated in multispecies biofilms. This suggests that these patterns reflect underlying, species-specific cellular characteristics that lead to different lysis efficiencies. A systematically decreased recovery of *S. oralis* and *A. naeslundii* using a given method, coupled with the enhanced recovery of *P. gingivalis*, distorts the apparent community structure in ways that could be misinterpreted as biological variation rather than recognized as technical artifacts.

The unexpectedly highest recovery of *P. gingivalis* with Method 3 may be attributed to the polysaccharide capsule enclosing these cells, which provides a physical barrier and mediates coaggregation with other species (Rosen and Sela 2006, Gerits et al. 2017). In addition, the gene expression of *P. gingivalis* differs considerably between planktonic and biofilm states (Lo et al. 2009). Especially multispecies oral biofilms facilitate various synergistic and cross-protective mechanisms (Lv et al. 2025), and *P. gingivalis* biofilms have been reported to exhibit markedly decreased susceptibility to bactericidal agents, compared to planktonic cells (Larsen 2002, Gerits et al. 2017). These factors may impede chemical and enzymatic lysis strategies for this species in biofilms, while favouring lysis through mechanical shear with the bead beating approach employed in Method 3. Apart from this distinction, Methods 2 and 3 performed largely equivalent, despite the manufacturer’s design of Method 3 (PowerBiofilm kit) specifically for biofilm samples. Notably, only Methods 1 and 2 incorporate Proteinase K digestion, which may disrupt cell aggregates more effectively by degrading proteinaceous adhesion structures. In Method 3, this enzymatic step is presumably compensated for by the enhanced polysaccharide-dissolving lysis reagent, as indicated in the manufacturer’s specifications.

Kit-based DNA extractions are often preferred over phenol-chloroform-based methods for downstream PCR applications, since residual phenol can inhibit amplification reactions (Demeke and Jenkins 2010). However, the chances for carryover can be substantially reduced by phase-separating gels (Molbert et al. 2023), as employed in the present study. Conversely, silica-column-based commercial extraction methods are also susceptible to carryover of e.g. guanidine salts, ethanol, and other buffer or sample components, which can similarly inhibit PCR reactions (Jue et al. 2020, Molbert et al. 2023, Tang and McMillen 2025). To mitigate potential bias from any inhibitor carryover on species quantification, method-specific standard curves were generated for each extraction method, using DNA standards prepared from pure culture extractions obtained with the corresponding method. It is also noteworthy that oxygen exposure during DNA extraction of strictly anaerobic bacteria has been shown to enhance DNA degradation, resulting in reduced recovery of high-molecular weight DNA (Boycheva et al. 2024). Consequently, the DNA extraction methods employed in this study could potentially be improved by performing them in an anoxic environment, although the practical impact of oxygen-induced DNA degradation on the relatively short qPCR amplicons (<200 bp) remains unclear.

### Method-dependent variation in biofilm community structure

To compare the community structure obtained by each method, relative species abundances were calculated as the percentage of DNA mass for each species relative to the total detected DNA mass across all species within a sample. The presence of only a single *rpoB* copy per genome ensured proportionality between quantified DNA mass and genome copy number. Thus, species abundances represent the relative distribution of genome copies, which approximates cellular abundance. It should be noted that these may deviate when cells contain multiple genome copies during active replication, and that the method also does not distinguish live from dead cells.

**Figure 3:**
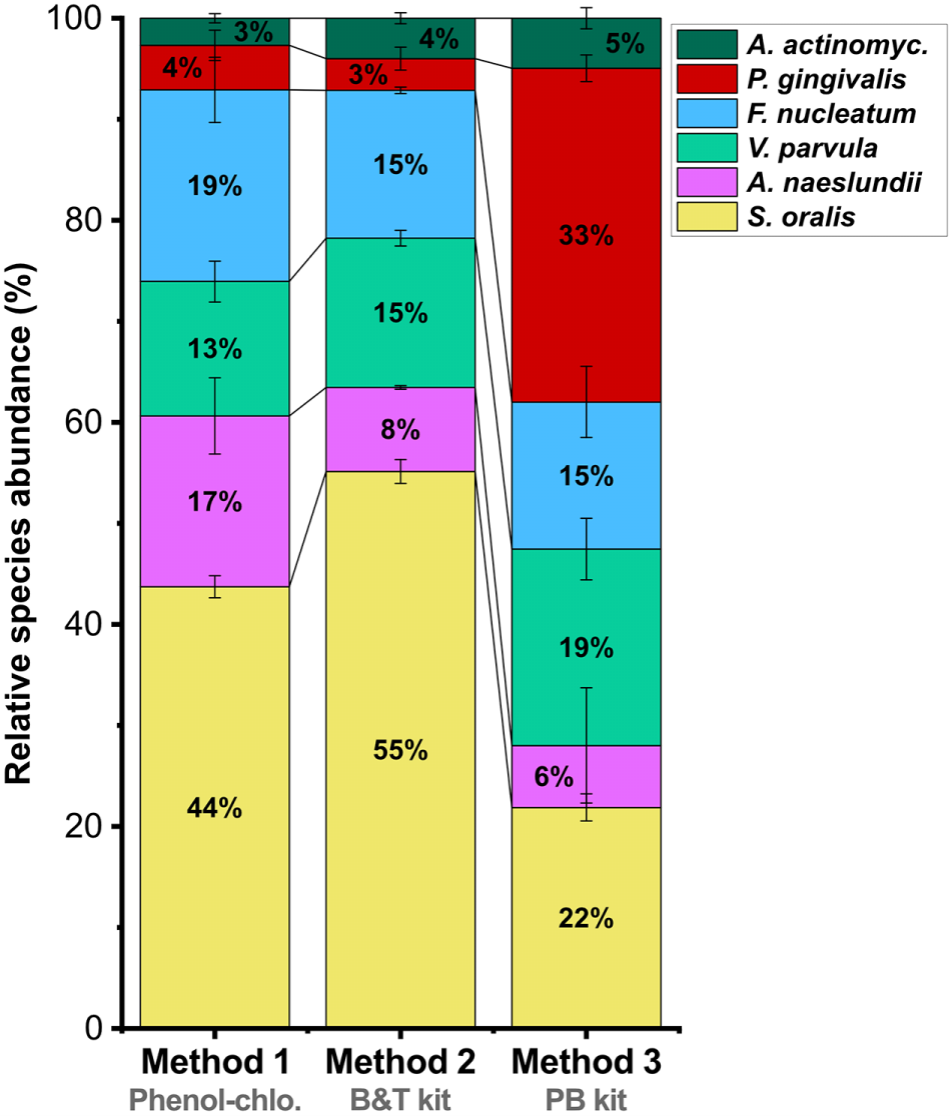
Microbial community composition of biofilms varies with extraction method. Relative species abundance (%) in multispecies biofilms, extracted with three different methods: Method 1 (Phenol-Chloroform), Method 2 (Commercial Kit—Blood and Tissue), and Method 3 (Commercial Kit—PowerBiofilm). Biofilms were cultivated anaerobically under static conditions on Ti-discs for 84 h. Quantification of individual species was performed by qPCR, using species-specific primers targeting the single-copy *rpoB* gene. Data represent mean percentages ± SD of biological replicates (*n* = 3).

As expected, the observed species-dependent extraction variances translated into markedly divergent community profiles, when expressed as relative abundances (Fig. 3). Methods 1 and 2 produced broadly comparable community structures, characterized by dominance of *S. oralis* (43.7% ± 1.1% and 55.1% ± 1.2%, respectively) and minimal representation of *P. gingivalis* (4.4% ± 1.5% and 3.1% ± 1.1%, respectively). In contrast, Method 3 yielded a fundamentally inverted profile, with *P. gingivalis* emerging as the dominant species (33% ± 1.3%) while *S. oralis* was reduced to 21.9% ± 1.3%. *Actinomyces naeslundii* exhibited substantial method-dependence, with a relative abundance of 16.9% ± 3.8% under Method 1 that decreased by more than half with Methods 2 and 3 (8.3% ± 0.2% and 6.1% ± 5.7%, respectively). In contrast, both middle colonizers *V. parvula* and *F. nucleatum* maintained more consistent representation (∼13%–19% and ∼14%–19%, respectively), while *A. actinomycetemcomitans* remained a minor constituent (∼3%–5%) across methods.

These compositional shifts demonstrate that the selection of DNA extraction methods can fundamentally alter interpretations of the microbiome. Depending solely on the method employed, the same biofilm appears dominated either by commensal early colonizers (predominantly *S. oralis*) or by pathogenic late colonizers (predominantly *P. gingivalis*). Although Method 1 achieved substantially higher total DNA yields than Method 2, the similar community profiles obtained with Methods 1 and 2 suggest that their shared chemical and enzymatic lysis strategies produce comparable species-specific extraction patterns. These community profiles, both characterized by *S. oralis* dominance, align with *in vivo* and *in vitro* observations of early oral biofilm maturation stages: Studies of natural biofilm formation on enamel and root surfaces (Nyvad and Kilian 1987, Diaz et al. 2006), as well as on titanium implant surfaces (Dieckow et al. 2024), have consistently documented predominance of streptococci in these biofilm communities. However, direct extrapolation to *in vivo* conditions should be approached with caution, given the comparatively less complex cultivation conditions of the model biofilms. More relevant, Kommerein et al. (2017) demonstrated in their static *in vitro* model, consisting of *S. oralis, A. naeslundii, P. gingivalis*, and *Veillonella dispar* grown on glass discs, that 48 h-old biofilms were heavily dominated by *S. oralis* with minimal relative abundances of *P. gingivalis*. Importantly, the authors corroborated their DNA-based species ratios with species-specific Fluorescence *In Situ* Hybridisation (FISH), providing independent validation that the observed *S. oralis* dominance reflects the true community composition of their model biofilms. The inverted community structure obtained with Method 3, dominated by *P. gingivalis* after 84 h cultivation, deviates substantially from these established ecological expectations, suggesting an extraction-driven artifact rather than a reflection of true community composition. Longer-term investigations of comparable static *in vitro* models have demonstrated that *P. gingivalis* remains a minor constituent for up to two weeks, before becoming the dominant species after three weeks of cultivation (Siddiqui et al. 2022). Importantly, in the absence of a comparable independent validation by e.g. FISH, the exact community composition that developed during the maturation period of our model biofilms remains unknown, as does the extraction method that reflects this most accurately. Identifying the most accurate method is, however, not required for the purpose of this study. We clearly demonstrate that the choice of extraction method can lead to fundamentally divergent apparent community profiles. This is sufficient to establish DNA extraction as a critical source of bias in defined biofilm models, irrespective of which method most accurately reflects the ‘‘true’’ composition.

The influence of DNA extraction methodology on (oral) microbiome characterization has been examined in several NGS studies, with variable conclusions. Rosenbaum et al. (2019) and Vesty et al. (2017) reported that bacterial diversity metrics did not differ significantly between extraction methods, despite substantial variation in DNA quality and yield. Conversely, Abusleme et al. (2014) and Yuan et al. (2012) demonstrated that the extraction method accounted for significant differences in apparent relative species abundances when examining mock communities with defined proportions of model species from the human (oral) microbiome. Notably, Abusleme et al. (2014) observed in the same study that the different extraction protocols had no influence on taxa prevalence when applied to natural supragingival plaque samples, but tended to affect the apparent relative abundance of individual taxa. Using a defined mock community comprising five oral species, Teng et al. (2018) compared extraction methods with emphasis on different lysis procedures and demonstrated substantial impacts on the community structure. Of particular relevance to the present findings, they observed a consistent overrepresentation of *S. oralis* when employing the same commercial kit used for Method 2 in this study. This concordance with the high *S. oralis* recovery with Method 2 observed here suggests a potential systematic overrepresentation of this species in the polymicrobial biofilm samples processed with this extraction approach.

Collectively, these findings underscore that DNA extraction method selection can profoundly influence apparent relative species abundances and the accurate representation of oral microbiota composition. While the overall diversity of natural samples appears to be more stable, potentially because the high taxonomic richness masks the extraction-bias of individual species, the effects can become substantially more pronounced in low-complexity communities. Given that *in vitro* biofilm models typically employ defined consortia comprising a limited number of representative species, such systems are particularly vulnerable to extraction bias. Consequently, careful selection and evaluation of DNA extraction methodology is crucial for these experimental approaches.

### Conclusion

The present study emphasizes that DNA extraction methodology represents a critical yet often underestimated variable in microbiome research. While there has been an increasing awareness of DNA extraction bias in other microbiome fields, particularly in gut microbiome research (Costea et al. 2017), this issue has received little attention in oral microbiome studies and has largely been overlooked in the context of *in vitro* biofilm models. The *in vitro* oral biofilm model examined here provides a particularly clear illustration of this bias and its impact on biofilms with defined consortia. The three methods examined here produced fundamentally different representations of similar biological samples, with species-specific biases that persisted from pure planktonic cultures to complex multispecies biofilms. The experimental DNA extraction approach determined whether the data from identical biofilm communities were dominated either by commensal early- or pathogenic late-colonising species, potentially leading to very different biological interpretations.

These findings have important implications for the interpretation and cross-study comparison not only of *in vitro* biofilm models, but also for other types of microbiome data. The observed systematic bias could widely compromise *in vitro* model validation and extrapolation to clinical scenarios. Given that these systems serve as critical tools for hypothesis testing alongside developing and evaluating novel therapeutic treatments or antimicrobial strategies, the compositional inversions observed here would lead to entirely different predictions regarding community dynamics, virulence potential, and therapeutic targets. The illustrated challenges extend to general microbiome research, as the field advances toward systems-level applications, increasingly demanding precise quantitative readouts (Noecker et al. 2016). Microbial interaction networks, metabolic models, and predictive frameworks for disease risk or treatment response all depend on accurate relative and absolute abundances. Especially precision medicine approaches that use microbiome signatures to assess disease progression and guide treatment decisions require diagnostic thresholds that are not compromised by biased or incomplete DNA extraction (Vandeputte et al. 2017).

The most appropriate DNA extraction method will ultimately depend on the specific research question, downstream application requirements, and the analytical objectives. For studies focused primarily on species presence/absence detection or qualitative community profiling, commercial kit-based methods provide sufficient performance, with the added advantages of being easy to implement and safe to use. For studies requiring quantification of absolute abundances, phenol-chloroform-based protocols that include comprehensive lysis procedures demonstrated higher and more consistent recovery across diverse cell types. However, the technical complexity of such protocols and their incorporation of hazardous compounds requires skilled and experienced users for correct execution (Molbert et al. 2023). As recommended by Teng et al. (2018), the extraction approach should generally be optimized for hard-to-lyse cells, when targeting communities of truly unknown composition. Fully resolving the question of which method most accurately reflects the community composition would require independent validation of biofilm species abundances by complementary approaches such as species-specific FISH. While this was beyond the scope of the present study, it will form the basis of future research.

We want to emphasize that the scope of this study was to highlight the risk and magnitude of DNA extraction-related bias, rather than to provide a comprehensive comparison across all relevant methods and taxa. This concern is particularly acute for *in vitro* models employing defined consortia of representative species, where such extraction bias affecting even a single taxon can fundamentally alter the apparent community structure. Researchers working with such systems should be aware that the methodological artefacts documented here may strongly affect their data and data interpretation, especially when relying on off-the-shelf DNA extraction solutions without extensive testing and optimization for their specific biofilm or microbiome samples.

## Supplementary Material

fnag066_Supplemental_File

## Data Availability

The original contributions presented in the study are included in the article/Supplementary material, further inquiries can be directed to the corresponding author/s.
