# Supplementary material for: The impact of DNA extraction methods on species quantification and apparent community composition of in vitro oral biofilms
Source: FEMS Microbiol Lett. 2026 Jun 8;373:fnag066. doi: 10.1093/femsle/fnag066 (PMC13285887; doi:10.1093/femsle/fnag066)
Supplement: fnag066_Supplemental_File [file fnag066_supplemental_file.pdf]

Supplementary material

Supplementary Table 1: Species-specific qPCR primer set used in this study.
Originally published in Reese et al. (2026).

Target species	Strain ID	Gene	Primer sequence	Amplicon size (bp)
S. oralis	NCTC 11427	*rpoB*	**F**-ACAGGCGAAATCAAGACCCAA<br>**R**-GCGGACCAACTGAGAAACG	114
A. naeslundii	ATCC 19039	*rpoB*	**F**-GTACCCACCGACACCATCTG<br>**R**-CGACGAGACCCACTACCTGA	198
V. parvula	NCTC 11810	*rpoB*	**F**-TGAGTGGTTCTTGGAGAGTGG<br>**R**-CGAAGTGGTGCTGCGTAAG	168
F. nucleatum	DSM 20482	*rpoB*	**F**-TCAACAACAACTCCTTTAGAACCA<br>**R**-GAGAAACAGAACCACCTGCTG	118
P. gingivalis	ATCC 33277	*rpoB*	**F**-TGGGGAGATAGTAGGTGACGA<br>**R**-CAGATTGTGGCAGAGAGGGG	130
A. actinomycetemcomitans	DSM 8324	*rpoB*	**F**-TTTCACATCGGAGGCTTTTTCAC<br>**R**-TATCGTGTATATCGGTGCGGAAG	136

Supplementary Table 2: DNA yield and quality of single-species extractions across all tested methods. Total yield in µg and DNA purity (absorbance ratios of 260/280 nm and 260/230 nm) were determined by NanoDrop. All values are given as the arithmetic mean ± standard error of the mean (n = 3).

	Species	Method 1	Method 2	Method 3
**Total DNA yield (µg)**	*S.oralis*	32.18 ± 0.89	4.66 ± 0.22	1.01 ± 0.12
DNA Purity				
**Abs 260 nm/280 nm**		2.02 ± 0.01	2.01 ± 0.02	1.98 ± 0.16
**Abs 260 nm/230 nm**		1.69 ± 0.03	1.46 ± 0.18	0.46 ± 0.11
**Total DNA yield (µg)**	*A.naeslundii*	12.19 ± 0.32	0.54 ± 0.02	0.6 ± 0.07
DNA Purity				
**Abs 260 nm/280 nm**		1.92 ± 0.02	2.03 ± 0.21	2.08 ± 0.23
**Abs 260 nm/230 nm**		1.38 ± 0.08	1 ± 0.11	0.06 ± 0.01
**Total DNA yield (µg)**	*V.parvula*	26.58 ± 0.6	2.55 ± 0.83	2.08 ± 0.13
DNA Purity				
**Abs 260 nm/280 nm**		1.95 ± 0.02	1.75 ± 0.09	1.94 ± 0.02
**Abs 260 nm/230 nm**		1.52 ± 0.05	0.95 ± 0.22	0.76 ± 0.27
**Total DNA yield (µg)**	*F.nucleatum*	28.58 ± 1.49	3.45 ± 1.39	1.78 ± 0.07
DNA Purity				
**Abs 260 nm/280 nm**		1.97 ± 0.01	1.91 ± 0.1	1.85 ± 0.07
**Abs 260 nm/230 nm**		1.5 ± 0.04	1.56 ± 0.12	0.56 ± 0.18
**Total DNA yield (µg)**	*P. gingivalis*	24.41 ± 0.94	2.72 ± 0.7	3.66 ± 0.04
DNA Purity				
**Abs 260 nm/280 nm**		1.92 ± 0.04	1.82 ± 0.02	1.89 ± 0.03
**Abs 260 nm/230 nm**		1.39 ± 0.08	1.29 ± 0.04	1.48 ± 0.14
**Total DNA yield (µg)**	*A. actinomycetem.*	24.27 ± 1.68	3.41 ± 1.37	2.93 ± 0.57
DNA Purity				
**Abs 260 nm/280 nm**		1.96 ± 0.01	1.88 ± 0.13	1.86 ± 0.03
**Abs 260 nm/230 nm**		1.53 ± 0.05	1.49 ± 0.18	1.1 ± 0.22

Supplementary Table 3: Statistical analysis for normal distribution and homogeneity of variance for DNA yields of single-species extractions across methods (for Figure 1).

Species	Method	Shapiro-Wilk Test		Levene's Test (W50)			
		p-value	Distribution	W50	df	p-value	Variance
S. oralis	M 1	**0.588**	Normal	1.696	2, 6	**0.261**	Homogeneous
	M 2	**0.295**	Normal				
	M 3	**0.080**	Normal				
A. naeslundii	M 1	**0.867**	Normal	2.512	2, 6	**0.161**	Homogeneous
	M 2	**0.832**	Normal				
	M 3	**0.099**	Normal				
V. parvula	M 1	**0.222**	Normal	0.512	2, 6	**0.623**	Homogeneous
	M 2	**0.189**	Normal				
	M 3	**0.675**	Normal				
F. nucleatum	M 1	**0.267**	Normal	0.661	2, 6	**0.550**	Homogeneous
	M 2	**0.154**	Normal				
	M 3	**0.783**	Normal				
P. gingivalis	M 1	**0.241**	Normal	0.747	2, 6	**0.513**	Homogeneous
	M 2	**0.303**	Normal				
	M 3	**0.406**	Normal				
A. actinomycet.	M 1	**0.165**	Normal	0.238	2, 6	**0.795**	Homogeneous
	M 2	**0.125**	Normal				
	M 3	**0.742**	Normal				

Shapiro-Wilk test for normality of distribution (n=3 per method).
Distribution assessment: Normal ($p > 0.05$), Non-normal ($p < 0.05$).
Levene's test for homogeneity of variance across methods.
Variance assessment: $p > 0.05$ means equal variances.
M = Method; **W50** = median-based Levene's statistic; **df** = degrees of freedom.
All assumptions for parametric ANOVA were met.

Supplementary Table 4: Statistical analysis of significant differences, using One-Way ANOVA, between DNA yields of single-species extractions across methods (for Figure 1).

Species	M. 1: Mean ± SD	M. 2: Mean ± SD	M. 3: Mean ± SD	F-statistic	df	p-value
S. oralis	32.18 ± 1.54	4.66 ± 0.37	1.01 ± 0.21	1027.17	2, 6	**< 0.001**
A. naeslundii	12.19 ± 0.56	0.54 ± 0.04	0.60 ± 0.13	1223.39	2, 6	**< 0.001**
V. parvula	26.58 ± 1.03	2.55 ± 1.45	2.08 ± 0.22	551.61	2, 6	**< 0.001**
F. nucleatum	28.58 ± 2.58	3.45 ± 2.4	1.78 ± 0.13	162.98	2, 6	**< 0.001**
P. gingivalis	24.41 ± 1.62	2.72 ± 1.21	3.67 ± 0.08	330.18	2, 6	**< 0.001**
A. actinomycet.	24.27 ± 2.90	3.41 ± 2.37	2.93 ± 0.99	88.88	2, 6	**< 0.001**

DNA yield expressed in µg (mean ± standard deviation, n=3 per method).
df = degrees of freedom.
All species showed significant differences among extraction methods ($p < 0.05$).

Supplementary Table 5: Statistical analysis: Post-hoc pairwise comparisons of DNA yields between extraction methods, using Tukey HSD test, for single-species extractions (for Figure 1).

Species	Comparison	Mean Diff. (µg)	95% CI		p-value	Significance
S. oralis	**M2** vs **M1**	-27.51	-29.82	-25.21	**< 0.001**	***
	**M3** vs **M1**	-31.17	-33.48	-28.86	**< 0.001**	***
	**M3** vs **M2**	-3.66	-5.96	-1.35	**0.007**	**
A. naeslundii	**M2** vs **M1**	-11.65	-12.48	-10.82	**< 0.001**	***
	**M3** vs **M1**	-11.59	-12.42	-10.75	**< 0.001**	***
	**M3** vs **M2**	0.06	-0.77	0.90	**0.970**	**ns**
V. parvula	**M2** vs **M1**	-24.03	-26.62	-21.44	**< 0.001**	***
	**M3** vs **M1**	-24.50	-27.08	-21.91	**< 0.001**	***
	**M3** vs **M2**	-0.47	-3.06	2.12	**0.848**	**ns**
F. nucleatum	**M2** vs **M1**	-25.13	-30.23	-20.03	**< 0.001**	***
	**M3** vs **M1**	-26.79	-31.90	-21.69	**< 0.001**	***
	**M3** vs **M2**	-1.66	-6.77	3.44	**0.603**	**ns**
P. gingivalis	**M2** vs **M1**	-21.70	-24.63	-18.77	**< 0.001**	***
	**M3** vs **M1**	-20.75	-23.67	-17.82	**< 0.001**	***
	**M3** vs **M2**	0.95	-1.98	3.88	**0.605**	**ns**
A. actinomycet.	**M2** vs **M1**	-20.87	-26.48	-15.26	**< 0.001**	***
	**M3** vs **M1**	-21.35	-26.96	-15.74	**< 0.001**	***
	**M3** vs **M2**	-0.48	-6.09	5.13	**0.962**	**ns**

M = Method; **CI** = Confidence interval.
Significance: *** $p < 0.001$, ** $p < 0.01$, * $p < 0.05$, ns = not significant.

Supplementary Table 6: Statistical analysis for normal distribution and homogeneity of variance for total DNA amounts per species (by qPCR) from multi-species biofilm extractions across methods (for Figure 2).

Species	Method	Shapiro-Wilk Test		Levene's Test (W50)			
		p-value	Distribution	W50	df	p-value	Variance
S. oralis	M 1	**0.763**	Normal	2.541	2, 6	**0.159**	Homogeneous
	M 2	**0.967**	Normal				
	M 3	**0.192**	Normal				
A. naeslundii	M 1	**0.978**	Normal	1.646	2, 6	**0.269**	Homogeneous
	M 2	**0.331**	Normal				
	M 3	**0.057**	Normal				
V. parvula	M 1	**0.760**	Normal	1.998	2, 6	**0.216**	Homogeneous
	M 2	**0.718**	Normal				
	M 3	**0.449**	Normal				
F. nucleatum	M 1	**0.661**	Normal	2.322	2, 6	**0.179**	Homogeneous
	M 2	**0.971**	Normal				
	M 3	**0.729**	Normal				
P. gingivalis	M 1	**0.493**	Normal	0.346	2, 6	**0.721**	Homogeneous
	M 2	**0.093**	Normal				
	M 3	**0.147**	Normal				
A. actinomycet.	M 1	**0.090**	Normal	0.687	2, 6	**0.539**	Homogeneous
	M 2	**0.232**	Normal				
	M 3	**0.065**	Normal				

Shapiro-Wilk test for normality of distribution (n=3 per method).
Distribution assessment: Normal ($p > 0.05$), Non-normal ($p < 0.05$).
Levene's test for homogeneity of variance across methods.
Variance assessment: $p > 0.05$ means equal variances.
M = Method**; W50** = median-based Levene's statistic; **df** = degrees of freedom.
All assumptions for parametric ANOVA were met.

Supplementary Table 7: Statistical analysis of significant differences, using One-Way ANOVA, between total DNA amounts per species (by qPCR) from multi-species biofilm extractions across methods (for Figure 2).

Species	M. 1: Mean ± SD	M. 2: Mean ± SD	M. 3: Mean ± SD	F-statistic	df	p-value
S. oralis	56.14 ± 13.79	25.34 ± 3.25	7.33 ± 0.69	27.24	2, 6	**0.001**
A. naeslundii	21.34 ± 4.80	3.76 ± 0.46	2.13 ± 2.13	36.72	2, 6	**< 0.001**
V. parvula	17.50 ± 6.83	6.92 ± 1.21	6.59 ± 1.63	6.82	2, 6	**0.029**
F. nucleatum	24.68 ± 8.31	6.73 ± 0.86	4.86 ± 1.17	15.17	2, 6	**0.005**
P. gingivalis	5.55 ± 1.70	1.54 ± 0.54	11.19 ± 2.02	28.99	2, 6	**0.001**
A. actinomycet.	3.34 ± 0.28	1.72 ± 0.04	1.63 ± 0.08	97.43	2, 6	**< 0.001**

DNA yield expressed as ng per biofilm (mean ± standard deviation, n=3 biological replicates per method).
df = degrees of freedom.
All species showed significant differences among extraction methods ($p < 0.05$).

Supplementary Table 8: Statistical analysis: Post-hoc pairwise comparisons of total DNA amounts per species (qPCR) between extraction methods, using Tukey HSD test, for multi-species biofilm extractions (for Figure 2).

Species	Comparison	Mean Diff. (ng)	95% CI		p-value	Significance
S. oralis	**M2** vs **M1**	-30.80	-51.32	-10.28	**0.009**	**
	**M3** vs **M1**	-48.80	-69.32	-28.29	**0.001**	***
	**M3** vs **M2**	-18.00	-38.52	2.51	**0.080**	**ns**†
A. naeslundii	**M2** vs **M1**	-17.58	-25.21	-9.96	**0.001**	***
	**M3** vs **M1**	-19.21	-26.84	-11.58	**0.001**	***
	**M3** vs **M2**	-1.62	-9.25	6.00	**0.797**	**ns**
V. parvula	**M2** vs **M1**	-10.58	-20.89	-0.27	**0.045**	*
	**M3** vs **M1**	-10.91	-21.22	-0.60	**0.040**	*
	**M3** vs **M2**	-0.33	-10.64	9.98	**0.995**	**ns**
F. nucleatum	**M2** vs **M1**	-17.96	-30.15	-5.76	**0.010**	*
	**M3** vs **M1**	-19.83	-32.03	-7.63	**0.006**	**
	**M3** vs **M2**	-1.87	-14.07	10.32	**0.887**	**ns**
P. gingivalis	**M2** vs **M1**	-4.01	-7.92	-0.11	**0.045**	*
	**M3** vs **M1**	5.63	1.73	9.54	**0.011**	*
	**M3** vs **M2**	9.65	5.74	13.55	**0.001**	***
A. actinomycet.	**M2** vs **M1**	-1.62	-2.04	-1.19	**< 0.001**	***
	**M3** vs **M1**	-1.70	-2.12	-1.28	**< 0.001**	***
	**M3** vs **M2**	-0.09	-0.51	0.34	**0.804**	**ns**

M = Method; **CI** = Confidence interval.
Significance: *** $p < 0.001$, ** $p < 0.01$, * $p < 0.05$, ns = not significant.
† Direct two-sample t-test for this comparison indicates significance: $t = 9.38$, $p = 0.001$.

References

Reese, J.-O., Lund, I. M. C., Haugen, H. J., Saragliadis, A., Lyngstadaas, S. P., & Linke, D. (2026). A dual-loop chemostat to investigate multi-species biofilms on implant surfaces under adjustable flow conditions. *Frontiers in Microbiology*, *17*, 1751315. https://doi.org/10.3389/fmicb.2026.1751315
